# Prognostic Value of Lymphocyte-to-White Blood Cell Ratio for In-Hospital Mortality in Infective Endocarditis Patients

**DOI:** 10.1155/2022/8667054

**Published:** 2022-02-25

**Authors:** Mengying Zhang, Qiuxia Ge, Tengfei Qiao, Yaman Wang, Xiaohong Xia, Xiang Zhang, Jun Zhou

**Affiliations:** ^1^Department of Laboratory Medicine, The First Affiliated Hospital of Nanjing Medical University, Nanjing, China; ^2^Branch of National Clinical Research Center for Laboratory Medicine, Nanjing, China; ^3^Department of Laboratory Medicine, Nanjing Lishui District Hospital of Traditional Chinese Medicine, Nanjing, Jiangsu, China

## Abstract

**Background:**

The prognosis of Infective endocarditis (IE) is poor, and we conducted this investigation to evaluate the worth of admission lymphocyte-to-white blood cell ratio (LWR) for prediction of short-term outcome in IE patients.

**Methods:**

We retrospectively assessed the medical records of 147 IE patients from January 2017 to December 2019. Patients were divided into the survivor group and nonsurvivor group. Univariate and multivariate analyses were applied to estimate the independent factors contribution to in-hospital death, and receiver-operator characteristic (ROC) curve was utilized to check the performance.

**Results:**

The levels of LWR (0.17 ± 0.08 vs. 0.10 ± 0.06) were significantly increased among the survivor group compared with the nonsurvivor group (*P* = 0.001). Multivariate analysis displayed that LWR (hazard ratio (HR): 1.755, 1.304–2.362, *P* < 0.001) was not interfered by other confounding factors for early death. Moreover, ROC analysis suggested that LWR (cutoff value = 0.10) performed the best among assessed indexes for the forecast of primary outcome (area under curve (AUC) = 0.750, 95% confidence interval (CI) = 0.634–0.867, *P* < 0.001, sensitivity = 70.0%, specificity = 76.4%), and the proportion of in-hospital mortality was remarkably inferior in patients with LWR > 0.10 than in those with LWR ≤ 0.10. (5.83% vs. 31.8%, *P* < 0.001).

**Conclusions:**

LMR is an independent, simple, universal, inexpensive, and reliable prognostic parameter to identify high-risk IE patients for in-hospital mortality.

## 1. Introduction

Infective endocarditis (IE) is associated with high morbidity and mortality and accompanied by severe complications [1]. Fever, embolic stroke, and heart failure are the most common symptoms; however, none of them is specific, making the diagnosis of the disease very difficult. Thus, the diagnostic criteria should include clinical manifestations, imaging, and laboratory results, and the modified Duke criteria is the most widely used [2, 3]. In the past, the detection technology and treatment methods were improved; however, the prognosis of IE remains poor (short-term and long-term mortality) [3, 4]. Therefore, to ameliorate the prognosis of IE, high-risk patients must be rapidly identified.

The diagnosis and prognosis biomarkers are widely used in many clinical diseases, such as endometrial cancer, gastric cancer, and rheumatoid arthritis [5–7]. Obesity, hemodialysis, and mean platelet volume (MPV) have clinical utility in IE [8–10]. However, a sole index could not truly reflect all possible conditions of patients. Recently, some studies tried to analyze the value of comprehensive indexes, such as lymphocyte and its related index in predicting prognosis of IE [11, 12], while the association of lymphocyte-to-white blood cell ratio (LWR) with short-term mortality in IE patients remains unknown. Therefore, we conducted this investigation to evaluate the value of admission LWR for prediction of short-term mortality in IE patients.

## 2. Patients and Methods

### 2.1. Patients and Data Collection

Patients (≥18 years old) with diagnosis of IE [3] were retrospectively analyzed from January 2017 to December 2019 in the First Affiliated Hospital of Nanjing Medical University (Nanjing, China). Patients meeting the criteria such as < 18 years old and incomplete data were excluded. This research was approved by the Ethics Committee of the First Affiliated Hospital of Nanjing Medical University (Nanjing, China) and followed the principles in the Declaration of Helsinki.

Patient's features and clinical characteristics were downloaded from medical records. Blood samples were taken when admission and measured within 2 hours. Blood routine indexes were analyzed using Sysmex XE 2100 analyzers (Sysmex, Japan), and chemistry data were analyzed using Beckman Coulter 5800 Clinical Chemistry analyzers (Beckman Coulter, Brea, USA). Follow-up results were available from medical records or telephone calls.

### 2.2. Outcomes

In-hospital mortality was the main outcome (all-cause death within 30 days).

### 2.3. Statistical Methods

Analyses were carried out with SPSS 21.0 (SPSS, Chicago, IL). Categorical data (*N*, proportions) and continuous variables (medians and interquartile or mean ± standard deviation) were compared by the chi-squared test, Mann–Whitney *U* test, or Student's *t*-test. Univariate analyses were applied to sieve probable predictors for in-hospital death. Furthermore, we included these factors (*P* value < 0.05 in the univariate analysis) into multivariate analyses to estimate their independent contribution to the primary outcomes. The performance of independent factors (LWR, NWR, and surgery) and lymphocyte and WBC was judged with receiver-operator characteristic (ROC) curve. *P* value < 0.05 was defined to demonstrate a significant difference.

## 3. Results

Table 1 provides the features of the IE patients. The age of the survivor group and nonsurvivor group was 50 ± 14.63 and 57 ± 11.74, respectively. Male occupied a large part of the two groups. The levels of WBC (9.28 ± 4.61 vs. 12.68 ± 5.79), neutrophil (7.25 ± 4.31 vs. 10.60 ± 5.66), neutrophil-to-WBC (NWR) (0.76 ± 0.10 vs. 0.82 ± 0.12), urea nitrogen (UREA) (6.45 ± 4.43 vs. 10.00 ± 6.23), creatinine (CREA) (69.7 (37.1, 944.2) vs. 83.8 (50.7, 902.0)), and neutrophil-to-lymphocyte (NLR) (5.02 (0.05, 72.53) vs. 9.33 (2.01, 64.52)) were significantly reduced, and LWR (0.17 ± 0.08 vs. 0.10 ± 0.06) was significantly increased among the survivor group compared with the nonsurvivor group (all *P* < 0.05) (Table 1). There was no significant difference in gender, lymphocyte, hemoglobin (HB), alanine aminotransferase (ALT), aspartate aminotransferase (AST), and platelet. Subsequently, multivariate analysis including age, WBC, neutrophil, LWR, NWR, UREA, CREA, NLR, and surgery were carried out. At last, LWR (hazard ratio (HR): 1.755, 1.304–2.362, *P* < 0.001), NWR (HR: 1.378, 1.145–1.659, *P* = 0.001), and surgery (HR: 6.146, 1.879–20.106, *P* = 0.003) were not interfered by other confounding factors for early death (Table 2).

In addition, Table 1 also suggested that there are 51 (34.7%) of the patients with admission positive blood culture. Among them, streptococci (63.8%) were the most frequent microorganisms, attended by *Staphylococcus* (25.5%) and others (10.7%).

**Figure 1:**
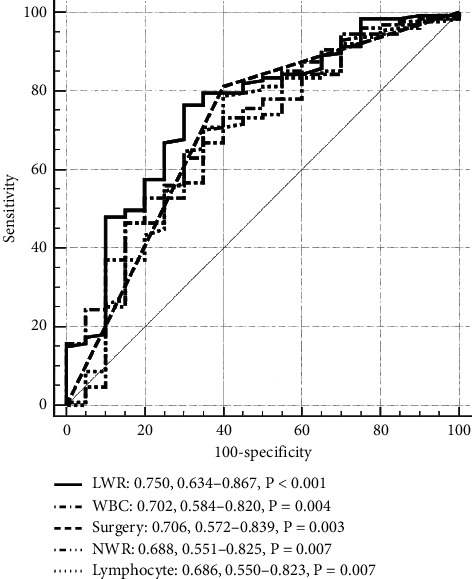
ROC curves of LWR in IE patients' in-hospital mortality prediction.

We applied ROC analysis to check the area under curve (AUC) to decide and compare the predictive value for independent factors, WBC, and lymphocyte. The results suggested that LWR (cutoff value = 0.10) had the highest performance for the forecast of primary outcome (AUC = 0.750, 95% confidence interval (CI) = 0.634–0.867, *P* < 0.001, sensitivity = 70.0%, specificity = 76.4%), followed by surgery (AUC: 0.706; 95% CI: 0.572–0.839, *P* = 0.003), WBC (AUC: 0.702; 95% CI: 0.584–0.820, *P* = 0.004), NWR (AUC: 0.688, 95% CI: 0.551–0.825, *P* = 0.007), and lymphocyte (AUC: 0.686; 95% CI: 0.550–0.823, *P* = 0.007) (Figure 1). The proportion of in-hospital mortality was remarkably inferior in patients with LWR >0.10 than in those with LWR ≤ 0.10. (5.83% vs. 31.8%, *P* < 0.001).

## 4. Discussion

In this work, a significant positive relationship between LWR and IE was discovered. Elevated LWR was an independent protection factor from in-hospital death for IE patients. In addition, LWR (at admission) was the most faultless for estimating in-hospital death.

The diagnosis of IE is difficult as the clinical presentations and laboratory data of IE are nonspecific, leading to the delayed diagnosis, treatment, and poor prognosis. The sooner high-risk patients are identified and appropriate intervention was taken, the better IE patient's prognosis will be [13, 14]. Previous researchers have evaluated the value of clinical and laboratory inflammation factors in prognosis [15, 16].

Lymphocyte, coming from lymphatic organs, is the important cellular component of the body's immune response function. It plays an important role in fighting against infections. Lymphocyte count varies from 1.1 × 10^9^/L to 3.2 × 10^9^/L in population; its proportion in total WBC is about 20%–50%. They usually can be elevated by virus infection. Inflammatory factors, derived from lymphocyte, including platelet-to-lymphocyte ratio (PLR), neutrophil-to-lymphocyte ratio (NLR), and LWR, are eagerly connected with the clinical results of patients [17]. Some studies suggested that lymphocyte and its related index, such as platelet-to-lymphocyte ratio (PLR) and neutrophil-lymphocyte ratio (NLR), were associated with the diagnosis and prognosis of IE [11, 12]. Our work also found that lymphocyte count was lower in the nonsurvivor group compared with the survivor group, while it was not significant. There are also some investigations that have assessed the relationship between LWR and several diseases [18, 19]. In a research of 336 COVID-19 patients, the investigators discovered that high LWR was related to the lowest increased risk of short-outcome (28-day mortality) [18]. For locally advanced gastric cancer patients with the capecitabine and oxaliplatin regimen, LWR with value ＜ 0.228 was independently associated with a low objective response rate and pathological remission rate [19]. Consistent with these results, our research also found that LWR could be served as an independent index to predict in-hospital death for IE patients. In addition, Zhao et al. reported that the performance of LWR was preferable to that of lymphocyte in predicting the outcome of advanced cancer patients receiving palliative care [20]. The current work also suggested that LWR has superior predictive ability than lymphocyte and is not interfered by other confounding factors.

Until now, we only found the relationship between low LWR and poor prognosis, and the potential mechanism is not well recognized and needs to be fully explored. In IE patients, various inflammatory factors have been investigated; however, this is the first time to analyze the value of LWR in predicting IE prognosis. Lymphocyte, especially T cells, plays an important part in immune surveillance and immune defense. T cells are promoted by the stimulation of antigen to differentiate into other subtypes, such as Th9, Th17, and tumor-infiltrated follicular helper, and secrete various cytokines. In turn, these cytokines may affect the distribution and survival of lymphocyte. Thus, lymphocyte and LWR level may act as a prognosis marker.

There were some limitations as follows. As a retrospective and single center research, the sample is small and selection bias is inevitable. Second, we did not confirm our results in another cohort. Therefore, a prospective, multicenters, and big sample cohort will be needed in the future.

## 5. Conclusion

LWR is an independent, simple, universally inexpensive, and reliable prognostic parameter to identify high-risk IE patients for in-hospital mortality.

## Figures and Tables

**Table 1:** Clinical characteristics of study population.

Variable	Nonsurvivor group, *N* = 20	Survivor group, *N* = 127	*P* value
Age (years)	57 ± 11.74	50 ± 14.63	0.044
Gender (male, *n* (%))	16 (80.0%)	86 (67.7%)	0.271
WBC (×10^9^/L)	12.68 ± 5.79	9.28 ± 4.61	0.004
Lymphocyte (×10^9^/L)	1.12 ± 0.93	1.36 ± 0.61	0.131
Neutrophil (×10^9^/L)	10.60 ± 5.66	7.25 ± 4.31	0.002
LWR	0.10 ± 0.06	0.17 ± 0.08	0.001
NWR	0.82 ± 0.12	0.76 ± 0.10	0.018
HB (g/L)	102.70 ± 13.75	106.62 ± 20.71	0.415
PLT (×10^9^/L)	175.40 ± 105.34	202.01 ± 111.37	0.319
ALT	22.7 (3.9, 2433.3)	24.1 (3.0, 280.2)	0.468
AST	27.5 (13.7, 3654.2)	24.2 (9.1, 218.6)	0.360
UREA	10.00 ± 6.23	6.45 ± 4.43	0.002
CREA	83.8 (50.7, 902.0)	69.7 (37.1, 944.2)	0.028
PLR	200.61 ± 132.95	166.85 ± 106.52	0.205
NLR	9.33 (2.01, 64.52)	5.02 (0.05, 72.53)	<0.001

Etiology			
*Staphylococcus*, *n* (%)	1 (5.00%)	11 (8.67%)	0.581
*Streptococcus*, *n* (%)	6 (30.00%)	24 (18.90%)	0.255
Others, *n* (%)	1 (5.00%)	8 (6.30%)	0.823
Culture negative, *n* (%)	12 (60.00%)	84 (66.14%)	0.595
Surgery (%)	40.00%	81.11%	<0.001

ALT, alanine aminotransferase; AST, aspartate aminotransferase; CREA, creatinine; HB, hemoglobin; LWR, lymphocyte-to-white blood cell ratio; NLR, neutrophil-to-lymphocyte ratio; NWR, neutrophil-to-WBC ratio; PLR, platelet-to-lymphocyte ratio; UREA, urea nitrogen; WBC, white blood cell.

**Table 2:** Multivariable logistic regression of in-hospital mortality for patients with infective endocarditis.

Variables	HR	95% CI	*P* value	Forest plot
LWR	1.755	1.304–2.362	<0.001	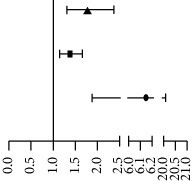
NWR	1.378	1.145–1.659	0.001	
Surgery	6.146	1.879–20.106	0.003	

CI, confidence interval; HR, hazard ratio; LWR, lymphocyte-to-white blood cell ratio; NWR, neutrophil-to-WBC ratio.

## Data Availability

The data used to support the findings of this study are available from the corresponding author upon request.

## Abbreviations

ALT: Alanine aminotransferase
AST: Aspartate aminotransferase
AUC: Area under curve
CI: Confidence interval
CREA: Creatinine
HB: Hemoglobin
HR: hazard ratio
IE: Infective endocarditis
LWR: lymphocyte-to-white blood cell ratio
MPV: Mean platelet volume
NLR: Neutrophil-to-lymphocyte ratio
NWR: Neutrophil-to-WBC ratio
PLR: Platelet-to-lymphocyte ratio
ROC: Receiver operator characteristic
UREA: Urea nitrogen
WBC: White blood cell.

## Additional Points

What is known about the topic? Infective endocarditis (IE) is related with severe complications and high mortality; however, predictive factors for prognosis, including short-term and long-term mortality of IE, remain poor. What does the study add? LWR is an independent, simple, universally, inexpensive, and reliable prognostic parameter to identify high-risk IE patients for in-hospital mortality.
